# *O*-desmethylangolensin inhibits the proliferation of human breast cancer MCF-7 cells by inducing apoptosis and promoting cell cycle arrest

**DOI:** 10.3892/ol.2013.1601

**Published:** 2013-10-01

**Authors:** EUN JEONG CHOI, GUN-HEE KIM

**Affiliations:** Plant Resources Research Institute, Duksung Women’s University, Tobong-ku, Seoul 132-714, Republic of Korea

**Keywords:** *O*-desmethylangolensin, breast cancer, apoptosis, cell cycle arrest

## Abstract

The aim of the present study was to investigate the anticancer effect of *O*-desmethylangolensin (*O*-DMA) by assessing cell proliferation, apoptosis and cell cycle distribution, as well as exploring the mechanisms underlying these effects in breast carcinoma MCF-7 cells. The cells were exposed to *O*-DMA (5–200 μM) for 24, 48 and 72 h. The results revealed that cell proliferation was significantly inhibited in a dose-dependent manner following treatment for 48 and 72 h, but not after 24 h, and resulted in the significant induction of apoptosis and the promotion of cell cycle arrest at the G_1_/S and G_2_/M phases. To elucidate these effects of *O*-DMA, the expression levels of cell cycle regulators were measured in the cells exposed to *O*-DMA at 150 μM for 72 h. Of the G_1_/S phase-related proteins, *O*-DMA modulated the cyclin-dependent kinases (CDKs), with a decrease in CDK2 and CDK4 and an increase in CDK6, and downregulated cyclin D and E. With respect to the G_2_/M-related proteins, *O*-DMA caused a reduction in CDK1, together with a slight increase in cyclin A and B. In addition, *O*-DMA downregulated p21^Cip1^ and p27^Kip1^, but not p16^INK4a^ and p15^INK4b^, and interacted with the CDK6-cyclin D and CDK1-cyclin B complexes. In conclusion, these results indicate for the first time that the regulation of the CDK4/6-cyclin D and CDK1-cyclin B complexes may participate in the anticancer activity pathway of *O*-DMA in MCF-7 cells.

## Introduction

*O*-desmethylangolensin (*O*-DMA) is one of the major metabolites of the isoflavone daidzein, and is produced by intestinal bacteria. *O*-DMA was first identified in 1986 (1), and 80–90% of the population produce this metabolite (2–5).

Although several researchers have speculated that the metabolites of isoflavones may be biologically important (6–8), few studies have been conducted. Epidemiological studies have led to the acceptance of the theory that isoflavones provide protection against breast cancer (9,10). Experimental studies have demonstrated that the daidzein precursor of *O*-DMA shows activity against various cancers, including breast cancer. Daidzein exhibits antiproliferative activity in breast cancer through the induction of apoptosis and cell cycle arrest (11–13). Moreover, as we reported previously (14), daidzein exerts its anticancer effects in human breast cancer cells via cell cycle arrest at the G_1_ and G_2_/M phases.

The present study investigated the possible anticarcinogenic effects of *O*-DMA through the inhibition of cell proliferation and cell cycle arrest in human breast carcinoma MCF-7 cells, which are regarded as an *in vitro* breast cancer model.

## Materials and methods

### Cell culture and O-DMA treatment

Human breast MCF-7 cells were purchased from the Korean Cell Line Bank (Seoul, Korea). MCF-7 were routinely maintained in RPMI-1640 (Sigma-Aldrich, St. Louis, MO, USA) supplemented with 10% fetal bovine serum and antibiotics (50 U/ml penicillin and 50 μg/ml streptomycin; Sigma-Aldrich) at 37°C in a humidified atmosphere containing 5% CO_2_. The synthesized *O*-DMA was a gift from Dr Lee (Department of Chemistry, Duksung Women’s University, Seoul, Republic of Korea) and dissolved in dimethyl sulfoxide (DMSO; final concentration, 0.1% in medium).

### 3-(4,5-Dimethylthiazol-2-yl)-2,5-diphenyltetrazolium bromide (MTT) assay

MCF-7 cell proliferation was assessed using the MTT assay. The cells were plated at a density of 1×10^5^ cells/well in a 96-well tissue culture plate (Corning Inc., Corning, NY, USA) and incubated at 37°C for 24 h. The plated cells were then treated with 5–200 μM *O*-DMA for 24, 48 and 72 h. Next, the plated cells were incubated with MTT (Sigma-Aldrich; final concentration, 0.5 mg/ml) for 4 h at 37°C. Subsequent to discarding all the medium from the plates, 100 μl DMSO was added to each well. The plates were placed at room temperature for 5 min with agitation, so that complete dissolution of the formazan was achieved. The absorbance of the MTT formazan was determined at 540 nm by a UV spectrophotometric plate reader (Emax; Molecular Devices, Sunnyvale, CA, USA). The IC_50_ value (the concentration of the extract required to inhibit cancer cell growth by 50% of the control level, which was cells treated with 0.1% DMSO) was estimated from the plot.

### Cell cycle distribution and detection of apoptosis

Cell cycle distribution and apoptosis were determined by fluorescence-activated cell sorting (FACS) analysis using propidium iodide (PI) staining to measure the DNA content. The MCF-7 cells were plated at a density of 5×10^5^ cells/well in a 6-well tissue culture plate (Corning Inc.) and incubated at 37°C for 24 h. Next, the cells were treated with 50, 150 and 200 μM *O*-DMA for 72 h. The cells were then harvested, washed with cold phosphate-buffered saline (PBS) and processed for the cell cycle analysis. Briefly, the cells were fixed in absolute ethanol and stored at −20°C for further analysis. The fixed cells were centrifuged at 800 × g and washed with cold PBS twice. RNase A (final concentration, 20 μg/ml; Sigma-Aldrich) and PI staining solution (final concentration, 50 μg/ml) were added to the cells and incubated for 30 min at 37°C in the dark. The cells were analyzed using a FACSCalibur instrument (BD Biosciences, San Jose, CA, USA) equipped with CellQuest 3.3 software.

For the detection of apoptosis, the cells were treated and harvested as aforementioned. As an apoptosis biomarker, phosphatidylserine, which is located on the cytoplasmic surface of the cell membrane, was detected using the Annexin V-FITC Apoptosis Detection kit (Calbiochem, EMD Chemicals Inc., Darmstadt, Germany). Annexin V and PI solution were added to the cell preparations and incubated for 25 min in the dark. Binding buffer (400 μl) was then added to each well and the samples were analyzed by flow cytometry.

### Immunoblotting and immunoprecipitation assay

The MCF-7 cells were plated at a density of 7.5×10^5^ cells/well in a 60-well culture plate and incubated at 37°C for 24 h. Next, the cells were treated with 50, 150 and 200 μM *O*-DMA for 72 h, then lysed in radioimmunoprecipitation assay buffer [1% NP-40, 150 mM NaCl, 0.05% sodium deoxycholate, 1% sodium dodecyl sulfate (SDS) and 50 mM Tris; pH 7.5) containing protease inhibitor mixture (Bio-Rad Laboratories, Hercules, CA, USA) for 1 h at 4°C. The supernatant was centrifuged at 13,000 × g and the protein concentration was determined using Bradford Protein Assay kit II (Bio-Rad Laboratories). Proteins (25 μg/well) denatured with sample buffer were separated by 10–12% SDS-polyacrylamide gel. The proteins were then transferred onto nitrocellulose membranes (0.45 μm). The membranes were blocked with a 1% bovine serum albumin solution for 3 h and washed twice with PBS containing 0.2% Tween-20, then incubated with primary antibodies overnight at 4°C. Rabbit polyclonal CDK1, goat polyclonal CDK2 and CDK4, rabbit polyclonal CDK6, mouse monoclonal cyclin A and D, rabbit polyclonal cyclin B and E, mouse monoclonal p15^INK4b^, p16^INK4a^, p18^INK4c^ and p19^INK4d^, rabbit polyclonal p21^Cip1^, p27^Kip1^ and p57^Kip2^ and goat polyclonal β-actin were purchased from Santa Cruz Biotechnology, Inc. (Santa Cruz, CA, USA) and used to probe the separate membranes. The following day, the immunoreaction was continued with the the respective secondary antibodies (goat anti-mouse IgG-HRP, goat anti-rabbit IgG-HRP, donkey anti-goat IgG-HRP, Santa Cruz Biotechnology Inc. after washing for 2 h at room temperature. The specific protein bands were detected by an Opti-4CN Substrate kit (Bio-Rad Laboratories). The relative intensity of the bands was analyzed by Quantity One Software (Bio-Rad Laboratories).

For the CDK-cyclin binding assay, the cells was treated and lysed as described in the immunoblotting section. The cell lysates (250 μg) were then incubated with the primary antibodies overnight at 4°C. The immune complexes were collected by incubation with protein A/G-plus agarose beads (Santa Cruz Biotechnology, Inc.) for 90 min at 4°C. The precipitates were washed with lysis buffer, denatured in sample buffer and analyzed by immunoblotting as described previously.

### Statistical analyses

All the experiments were repeated four times. Data are presented as the mean ± SD (n=4–7), with the exception of the reactive oxygen species scavenging and antiproliferative activity data, which are expressed as percentages compared with the vehicle-treated control cells, which were arbitrarily assigned 100%. Data were analyzed by one-way analysis of variance followed by Dunnett’s multiple comparison test using SigmaStat (Jandel Scientific, San Rafael, CA, USA). P<0.05 was considered to indicate a statistically significant difference.

## Results

### Inhibition of cell proliferation by O-DMA

**Figure 1:**
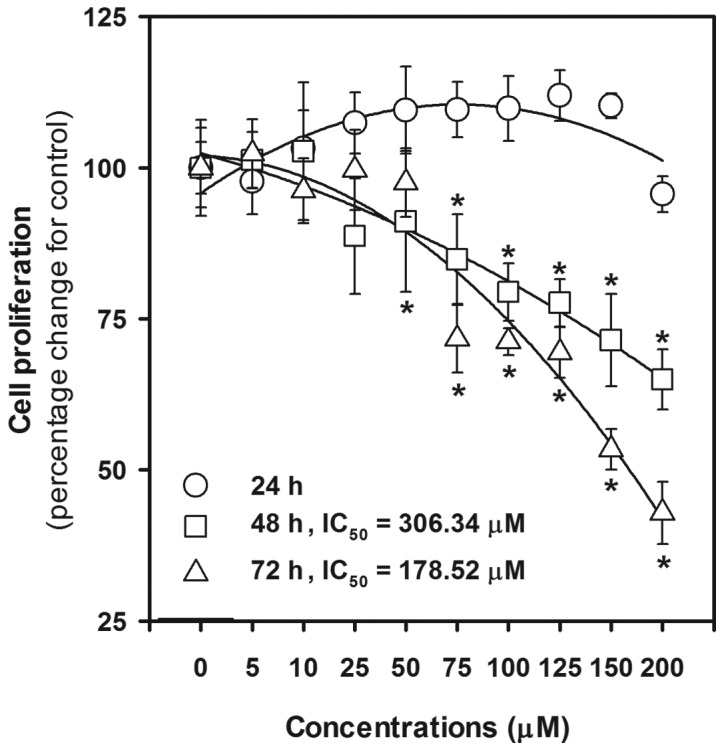
Antiproliferative activity of *O*-desmethylangolensin (*O*-DMA). The MCF-7 cells were exposed to either the vehicle or *O*-DMA (5–200 μM) and incubated for 24, 48 and 72 h. All data are reported as the percentage changes in comparison with the vehicle-only group, which was arbitrarily assigned 100% viability. ^*^P<0.05, significantly different from the vehicle-only group [0.1% dimethyl sulfoxide (DMSO) in medium].

The effect of *O*-DMA on the proliferation of the human breast cancer MCF-7 cells was measured using an MTT assay. Treatment of the MCF-7 cells with *O*-DMA for 24 h increased cell proliferation by up to 10%, however, this difference was not significant (Fig. 1). *O*-DMA significantly decreased cell proliferation after 48 and 72 h in a dose- and time-dependent manner (306.34 and 178.52 μM at IC_50_ for 48 and 72 h, respectively; P<0.05). Cell proliferation was decreased by 55.15% compared with the controls after treatment with 200 μM *O*-DMA for 72 h.

### O-DMA induces cell cycle arrest and apoptosis

**Figure 2:**
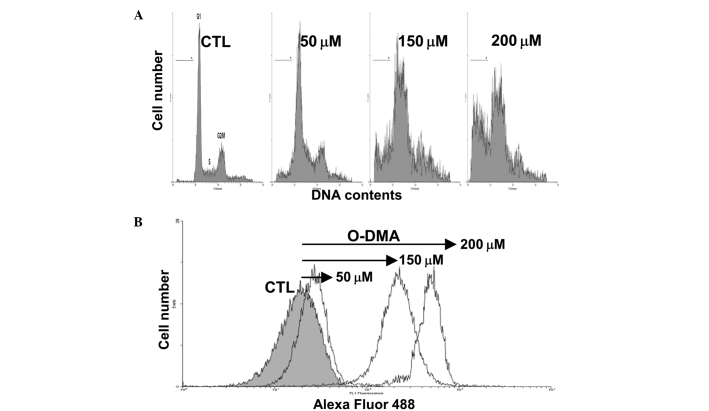
*O*-desmethylangolensin (*O*-DMA) induces (A) cell cycle arrest and (B) apoptosis. Cell cycle distribution and apoptosis were determined by fluorescence-activated cell sorting (FACS) analysis using propidium iodide (PI) staining to measure DNA content and an Annexin V assay, respectively. MCF-7 cells were exposed to either vehicle [0.1% dimethyl sulfoxide (DMSO) in medium] or *O*-DMA (50, 150 and 200 μM) for 72 h.

The MCF-7 cells were treated with 50, 150 and 200 μM *O*-DMA for 72 h, and DNA synthesis arrest was determined by FACS analysis (Fig. 2). *O*-DMA-induced cell cycle arrest in the G_1_/S and G_2_/M phases was observed in a dose-dependent manner. Additionally, a sub-G_1_ peak was observed, indicating *O*-DMA-induced apoptosis. A significant increase in the number of sub-G_1_ phase cells was observed following exposure to 50, 150 and 200 μM *O*-DMA (8.4, 17.1 and 37.8% of the cell population, respectively, compared with the controls, 1.3%; P<0.05).

### Modulation of G_1_/S and G_2_/M checkpoint regulators by O-DMA

**Figure 3:**
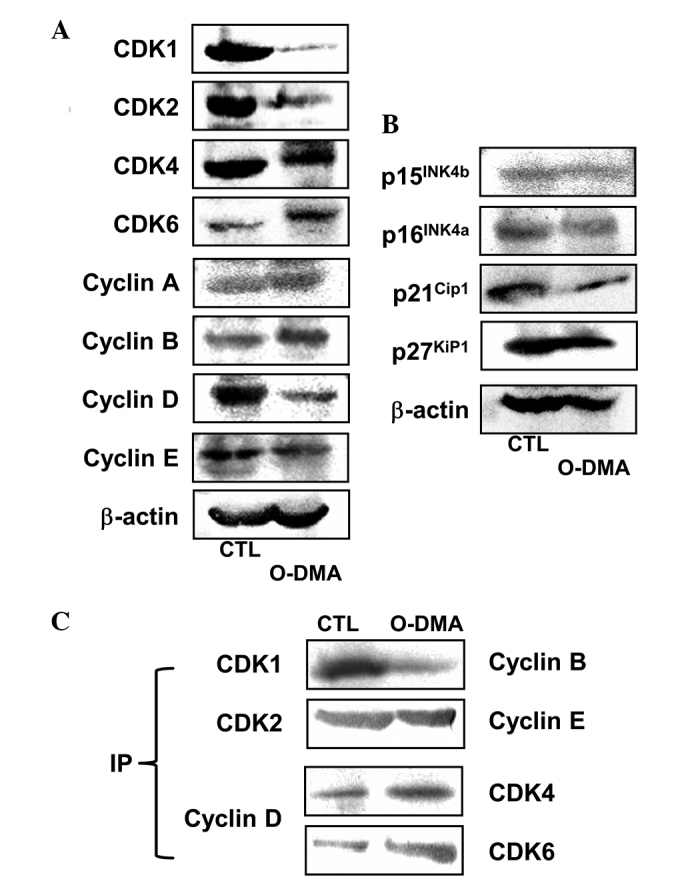
Modulation of genes related to cell cycle checkpoints by *O*-desmethylangolensin (*O*-DMA). (A and B) Protein expression and (C) binding assay of cyclin-dependent kinases (CDKs) and cyclins [IP (immunoprecipitation)]. MCF-7 cells were exposed to either vehicle [0.1% dimethyl sulfoxide (DMSO) in medium] or *O*-DMA (15 μM) for 72 h.

To investigate the effect of *O*-DMA on the expression of the cell cycle regulatory proteins, the MCF-7 cells were treated with 150 μM *O*-DMA for 72 h (Fig. 3A). The *O*-DMA treatment resulted in marked reductions in the expression of CDK1 and CDK2, given as expression densities; i.e., the ratio of each protein to β-actin was <5% compared with the control level. CDK4 and CDK6 expression changed in opposite fashions, with CDK4 decreased by 76.9% and CDK6 increased by 152.3% compared with the controls (P<0.05). *O*-DMA increased the expression of cyclins A and B, with only cyclin B increased significantly by 118.7% compared with the control (P<0.05). By contrast, the expression of cyclins D and E decreased following exposure to *O*-DMA (by 14.2 and 46.7%, respectively, compared with the control level; P<0.05).

*O*-DMA treatment significantly altered the expression of members of the INK4 and CIP/KIP families compared with vehicle-treated MCF-7 cells (P<0.05; Fig. 3B). *O*-DMA did not change the p16^INK4a^ and p15^INK4b^ expression levels, while p21^Cip1^ and p27^Kip1^ expression was decreased slightly. In addition, formation of the CDK4/6-cyclin D complex was increased in the MCF-7 cells in response to *O*-DMA, while the CDK1-cyclin B complex levels were decreased (Fig. 3C).

## Discussion

Although there are only minor differences in the structures of the isoflavones, genistein and daidzein, and their metabolites, *O*-DMA and equol, the anticancer effects and mechanisms are diverse. The present study first examined the antiproliferative effect of *O*-DMA on human breast cancer MCF-7 cells. *O*-DMA significantly decreased cell proliferation in a dose-dependent manner following exposure for 48 and 72 h. This was consistent with previous studies that demonstrated that daidzein inhibits the growth of human breast carcinoma cells (11–14). Similar to phytoestrogen, daidzein, the precursor of *O*-DMA, may possess biphasic activity (inhibitory at high concentrations and stimulatory at low concentrations) (15,16). Although *O*-DMA has only a weak affinity for the estrogen receptor (17–19), the exposure to *O*-DMA in the present study resulted in slightly increased MCF-7 cell growth after only 24 h. Thus, *O*-DMA may be a more promising anticancer candidate than its precursor.

To further understand the mechanisms underlying the anticancer activity of *O*-DMA, apoptosis induction and the cell cycle distribution of the MCF-7 cells exposed to 50, 150 and 200 μM *O*-DMA for 72 h were analyzed in the present study. Similar to the cell proliferation data, *O*-DMA induced significant apoptosis and cell cycle arrest at the G_1_/S and G_2_/M phases in a dose-dependent manner. Numerous studies have demonstrated that the majority of flavonoids induce G_1_ phase arrest in human cancer cells and that certain flavonoids inhibit the cell cycle at either the G_1_/S or G_2_/M phases in various human cancer cells (20–22). In our previous study, daidzein caused cell cycle arrest in the G_1_/S or G_2_/M phases, depending on the cell line (14). Thus, cell cycle arrest at the G_1_/S and G_2_/M phases and apoptosis induction by *O*-DMA support the hypothesis that *O*-DMA has useful anticancer properties.

The cell cycle is divided into four distinct phases, G_1_, S, G_2_ and M, and is tightly controlled by catalytic complexes of CDKs/cyclins that coordinate internal and external signals at several key checkpoints. Activated CDK-cyclin complexes may be changed to an inactive state by binding to CDK inhibitory subunits (CKIs), which are divided into two classes, namely the CIP/KIP (p21^Cip1^, p27^Kip1^ and p57^Kip2^) and INK4 (p16^INK4a^, p15^INK4b^, p18^INK4c^ and p19^INK4d^) families (23,24). Cell cycle arrest leads to either the inhibition of proliferation or the activation of the apoptosis pathway (25,26). Therefore, the discovery of anticancer candidate compounds that may function to regulate CDKs, is a therapeutic strategy that may result in improved cancer therapies.

*O*-DMA decreased CDK4 and cyclin D expression in the present study, however, it also increased CDK6 expression. In addition, the expression levels of CDK2 and cyclin E were decreased by *O*-DMA. These data are consistent with the ability of *O*-DMA to arrest the cell cycle at the G_1_/S phase. G_1_ phase progression and G_1_/S phase transition are regulated by CDK2 and CDK4, which assemble with cyclin E and D. CDK4-cyclin D and CDK2-cyclin E act predominantly during the G_1_/S transition (27).

Activated CDK-cyclin complexes are inactivated by binding to CKIs. The CIP/KIP family has a preference for CDK2- and CDK4-cyclin complexes, and the INK4 family is specific for CDK4- and CDK6-cyclin complexes (28,29). *O*-DMA had effects on p21^Cip1^ and p27^Kip1^ of the CIP/KIP family in the present study. It has been previously reported that p21^Cip1^ promotes cell cycle arrest in response to a number of antiproliferative signals (30). Moreover, in the present study, *O*-DMA reduced the levels of CDK1 and significantly increased those of cyclin B, but not cyclin A. CDK1 is a catalytic subunit of the M phase promoting factor, which is activated at the G_2_/M transition and controls the onset of mitosis (31). A previous study demonstrated that CDK1, in combination with cyclin A and B, is critical for the G_2_/M phase transition (32). Moreover, the present study detected significantly increased levels of CDK4/6-cyclin D and decreased levels of CDK1-cyclin B. Based on these data, *O*-DMA acted on multiple cell cycle regulators, including affecting the interaction between the CDK4/6-cyclin D and CDK1-cyclin B complexes.

In conclusion, *O*-DMA may exert anticancer activity through the inhibition of cell proliferation and the induction of apoptosis in human breast cancer MCF-7 cells. Moreover, the present study indicates for the first time that the regulation of the CDK4/6-cyclin D and CDK1-cyclin B complexes may participate in the anticancer activity of *O*-DMA.
